# HIV risk taking and risk preventing behaviors in men who have sex with men and women: a respondent-driven sampling study in Shenzhen, China

**DOI:** 10.1186/1471-2334-14-S2-P26

**Published:** 2014-05-23

**Authors:** Rui Cai, Jin Zhao, Wende Cai, Lin Chen, Jan Hendrik Richardus, Sake J de Vlas

**Affiliations:** 1 Erasmus MC, University Medical Center Rotterdam, Rotterdam, the Netherlands

## Introduction

Men who have sex with men (MSM) has clearly become the predominant route of HIV transmission in China. Due to traditional cultures and values, many MSM have frequent sexual relationship with females. These men who have sex with men and women (MSMW) may bridge and facilitate an expansion of the HIV epidemic from MSM to the general heterosexual population.

## Methods

We quantified the burden of HIV/syphilis and studied patterns of risk taking and preventing behaviors in 107 MSMW, and compared that with 542 men who have sex with men only (MSMO) in a respondent-driven sampling survey in Shenzhen, China.

## Results

The HIV risk preventing behaviors such as uptake of HIV testing and other services and consistent condom use with male partners were comparable in MSMW and MSMO. However, HIV risk taking behaviors, in terms of having multiple anal sex partners, using illicit drugs, using alcohol before or during sex and having group sex, were more common among MSMW than among MSMO. Moreover, HIV prevalence was as high as 6% and consistent condom use was low: 41% with wives and 38% with girlfriends in MSMW.

## Conclusions

We conclude that there is risk of HIV transmission from MSMW to the general heterosexual population. Special efforts targeting MSMW are needed to reduce their HIV risk taking behaviors. Outreach education programs implemented by their peers who are behaviorally bisexuals may be an important strategy.

